# Supplementary figures and images for: Bioactive Compounds of Underground Valerian Extracts and Their Effect on Inhibiting Metabolic Syndrome-Related Enzymes Activities
Source: Foods. 2023 Feb 2;12(3):636. doi: 10.3390/foods12030636 (PMC9914926; doi:10.3390/foods12030636)

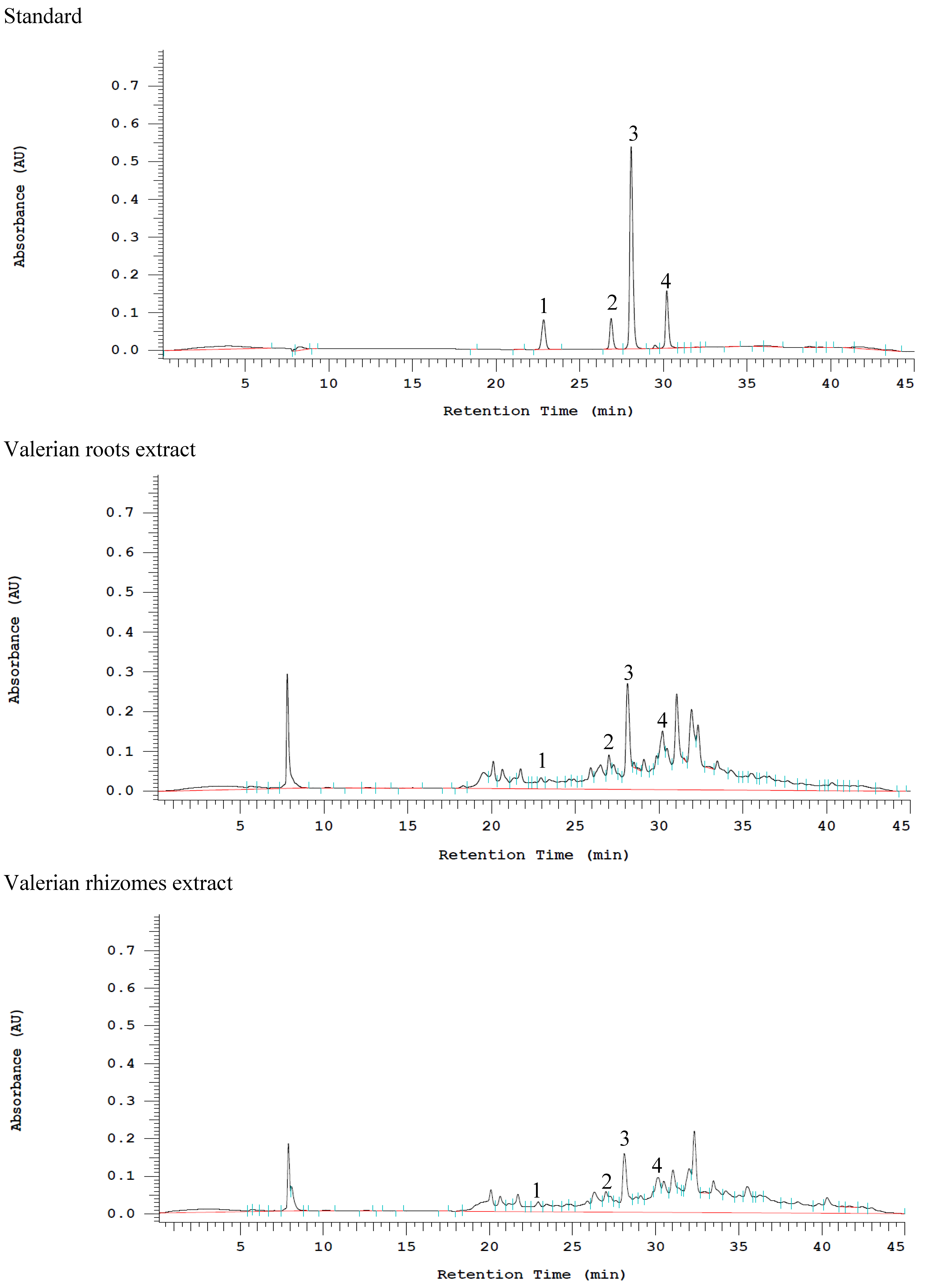

Supplement: Supplementary file 1 [file foods-12-00636-s001.zip › Figure S1-phenolic acids.tiff]

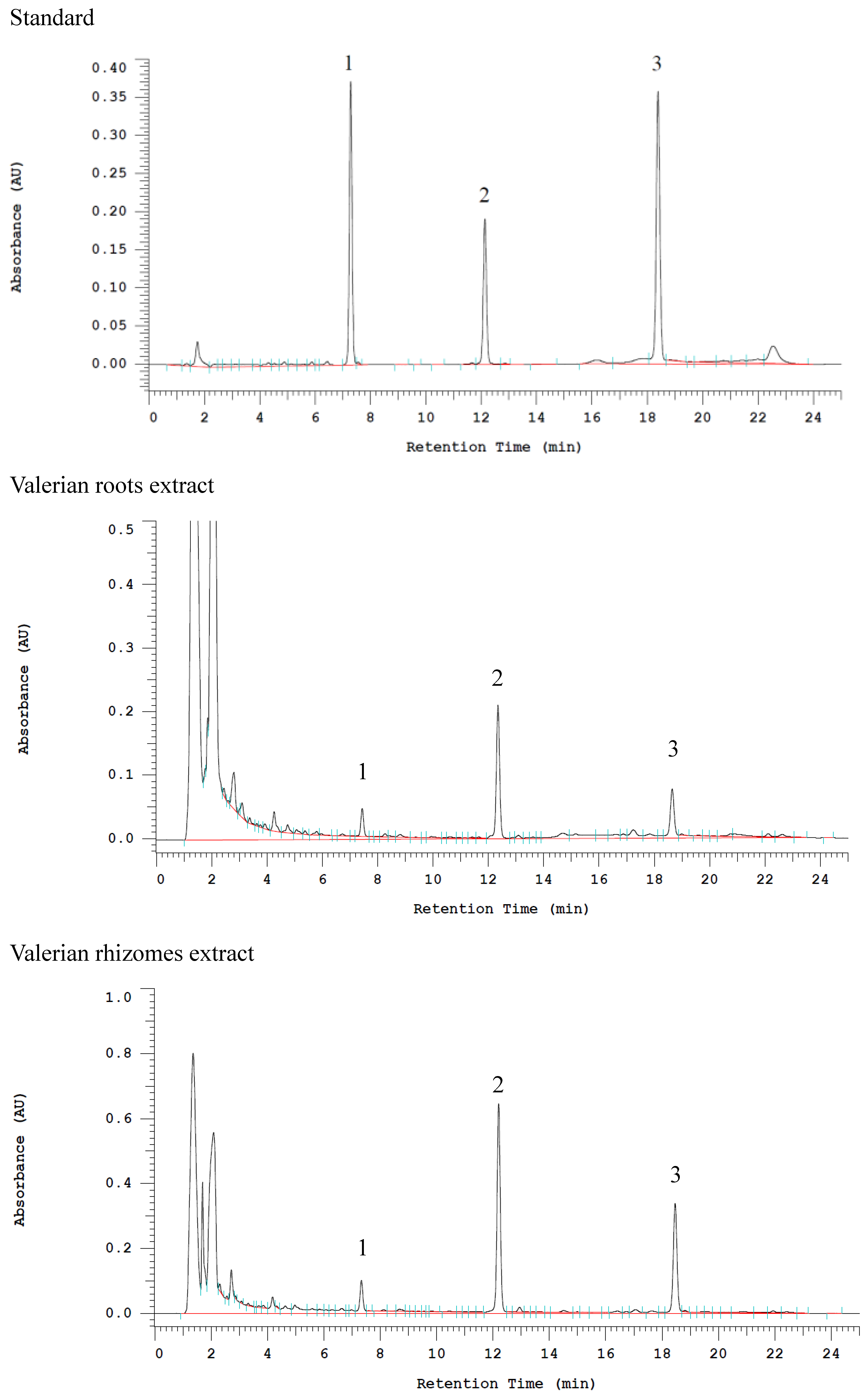

Supplement: Supplementary file 1 [file foods-12-00636-s001.zip › Figure S2-valerenic acid derivatives.tiff]
